# Salidroside and exercise performance in healthy active young adults – an exploratory, randomized, double-blind, placebo-controlled study

**DOI:** 10.1080/15502783.2024.2433744

**Published:** 2024-11-27

**Authors:** Neil A. Schwarz, Matthew T. Stratton, Ryan J. Colquhoun, Alexia M. Manganti, Margaux Sherbourne, Florian Mourey, Caitlyn C. White, Heather Day, Micaela C. Dusseault, Geoffrey M. Hudson, Christopher R. Vickery, Holly C. Schachner, Philip G. Kasprzyk, Jing-Ke Weng

**Affiliations:** a University of South Alabama, Exercise and Nutrition Research Group (ENRG), Department of Health, Kinesiology, and Sport, Mobile, AL, USA; b University of South Alabama, Department of Physiology and Cell Biology, Frederick C. Whiddon College of Medicine, Mobile, AL, USA; c Department of Research and Applications, Gnosis by Lesaffre, Lesaffre Group, Marcq-en-Baroeul, France; d Recombia Biosciences by Lesaffre, Bedford, MA, USA; e DoubleRainbow Biosciences Inc., Lexington, MA, USA; f Northeastern University, Institute for Plant-Human Interface, Boston, MA, USA; g Northeastern University, Department of Chemistry and Chemical Biology, Department of Bioengineering, and Department of Chemical Engineering, Boston, MA, USA

**Keywords:** Salidroside, rhodiola rosea, adaptogen, high-intensity interval exercise, oxygen consumption, golden root

## Abstract

**Background:**

Rhodiola rosea extract is purported to improve physical performance and support resilience to stress. Salidroside is considered to be one of the main constituents responsible for the ergogenic actions of R. rosea. However, R. rosea extract contains relatively little salidroside and cultivation of R. rosea is challenging as it is mainly found in high-altitude, cold regions. Additionally, the R. rosea plant is subject to conservation concerns because of its growing popularity. The purpose of this exploratory study was to evaluate the short-term effects of pure, biosynthetic salidroside supplementation on exercise performance, mood state, and markers of inflammation and muscle damage in healthy active young adults.

**Methods:**

Fifty participants (30 M, 20F; 21 ± 4 yrs; 173 ± 8 cm; 74 ± 13 kg) were randomly assigned to either salidroside (60 mg/day for 16 days) or placebo supplementation and underwent peak oxygen uptake (VO_2_ peak), intermittent time-to-exhaustion (TTE), and local muscular endurance assessments, along with mood state evaluations using the Profile of Mood States (POMS). Blood samples were analyzed for erythropoietin, myoglobin, creatine kinase-MM, and C-reactive protein.

**Results:**

Salidroside supplementation enhanced overall percent predicted oxygen uptake during high-intensity intermittent exercise (*p* < 0.01). An increase in serum myoglobin was observed 24 hours following exercise in the placebo group (*p* = 0.02) compared with baseline whereas no statistically significant increase was observed for the salidroside group indicating reduced exercise-induced muscle damage. Placebo group experienced a decrease in number of intervals performed during the TTE test (*p* = 0.03), and a decrease in friendliness (*p* < 0.01) and an increase in fatigue-inertia (*p* < 0.01) as reported by POMS. The salidroside group exhibited stable mood states and maintained performance levels during the time-to-exhaustion test.

**Conclusion:**

Salidroside supplementation may enhance oxygen utilization and mitigate exercise-induced muscle damage and fatigue, warranting further research on its long-term effects and potential as an adaptogen for active individuals.

## 1. Introduction

Proper nutrition is essential for optimal physical and mental performance, and overall well-being of active individuals [1]. To aid their nutritional intake, active individuals and athletes at all levels of sport commonly consume dietary supplements for various purposes such as improved exercise performance, recovery, and mood enhancement [2]. Adaptogens are one class of dietary supplements traditionally used to increase attentiveness and endurance during fatiguing/stressful activities and to enhance general resistance to physical or mental stress [3]. *Rhodiola rosea* (*R. rosea*) extract has gained attention as a potential adaptogen to support these outcomes [4]. *R. rosea* is a perennial flowering plant that grows in high-altitude, cold regions and has been utilized for centuries for its purported adaptogenic properties [3]. At least 140 known compounds in *R. rosea* extracts are believed to exert bioactivity upon ingestion with the most active considered to be salidroside, tyrosol, rosavin, and triandrin [3,5]. Of these, salidroside, a glucoside of tyrosol, is considered to be the primary bioactive component of *R. rosea* [6]; thus, further investigation of this particular bioactive in humans is needed.

To date, salidroside has only been studied using *in vitro* cell-based models and *in vivo* animal models. *In vitro* studies suggest salidroside acts as a 5′adenosine monophosphate-activated protein kinase (AMPK) activator resulting in enhanced glucose uptake and utilization and mitochondrial function [7,8]. In conjunction with enhanced substrate utilization, salidroside-induced AMPK activation was linked to improved endothelial function by promoting nitric oxide production via the AMPK/Phosphatidylinositol 3-kinase (PI3K)/Protein Kinase B (Akt)/endothelial nitric oxide synthase (eNOS) pathway which may result in improved blood distribution during intense exercise [9]. Further, salidroside-induced AMPK activation has been purported to have an anti-inflammatory effect on endothelial cells resulting in reduction of circulating inflammatory factors such as interleukin-6 (IL-6), interleukin-1β (IL-1β), and tumor necrosis factor-α (TNF-α) via inhibition of the nuclear factor (N)F-κB p65/NOD-like receptor protein 3 (NLRP3) signaling pathway [10]. In addition, salidroside has been shown to promote erythropoiesis and protection of erythroblasts potentially increasing ability to transport oxygen [11].

Multiple investigations involving salidroside in rodent models have shown improvements in exercise tolerance. In one study, salidroside displayed significant anti-fatigue activity in mice after 15 days of administration by prolonging swimming time accompanied by increases in muscle and liver glycogen and hemoglobin and a reduction in blood lactate [12]. Similarly, chronic administration of salidroside over four weeks also increased swimming time to exhaustion while reducing malondialdehyde (MDA), a metabolite of phospholipid peroxidation, and increasing antioxidant activity of superoxide dismutase (SOD), catalase (CAT) and glutathione peroxidase (GSH-Px; 13). Liver glycogen levels were also higher post-swimming compared with control suggesting enhanced liver glycogen stores or reduced liver glycogen metabolism during exercise [13]. In another study, salidroside reduced oxidative stress and prolonged swimming time in mice exposed to a hypoxic environment [14]. In addition to physical performance, several studies with salidroside described cognitive effects in animal models. Salidroside was shown to attenuate stress-induced depression in mice, presumably through interaction with pathways in the microglia, such as extracellular signal-regulated kinase (ERK)1/2, P38 mitogen-activated protein kinase (MAPK), and p65 NF-κB [15]. Salidroside was also shown to modulate neurological behavior in rats, exhibiting antidepressant-like effects in rats subjected to chronic mild stress [16]. Interestingly, another study reported that salidroside attenuates stress-induced binge eating in female rats [17].

The aforementioned rodent studies demonstrate the utility of salidroside in both physical performance and mental function warranting its investigation within the context of human health and function. Until now, a major limitation of studying the effect of salidroside supplementation in humans has been the lack of commercially available salidroside for dietary use. Thus, investigations in human participants up to this point have utilized *R. rosea* extract typically standardized to 1% salidroside. Ingestion of *R. rosea* extract has been shown to be useful for improvement of various exercise modalities; however, not all studies agree [18]. De Bock *et al*. [19] found acute ingestion of 200 mg of *R. rosea* extract (1% salidroside) was capable of augmenting endurance exercise capacity. A decrease in heart rate and perception of effort (RPE) was observed during submaximal exercise after ingestion of 3 mg/kg of *R. rosea* (1% salidroside) 60 minutes prior to exercise initiation [20]. Similarly, a 2014 study by Duncan *et al*., noted reductions in RPE and increases in pleasure following a 30-minute cycling bout at 70% of VO_2_max in recreationally trained males when compared to placebo (maltodextrin; 20). Furthermore, consuming 500 mg of *R. rosea* (1% salidroside) 30 minutes prior to exercise following three days of supplementation has previously resulted in increased mean power, anaerobic capacity, peak power, and total work throughout 3 × 15 second Wingate cycling tests in physically active females [21] in addition to greater mean concentric velocity at 75% of bench press 1-RM in resistance-trained males, despite a decrease in repetition to failure performance [22]. Thus, acute *R. rosea* ingestion may increase the ability to perform explosive tasks at the expense of total work when performing resistance exercise. Lastly, the effect of thirty days of *R. rosea* supplementation on muscle endurance and explosiveness in both rodents and human subjects was shown to be further augmented by acute caffeine consumption [23]. In rats, the combination of R. rosea and caffeine significantly increased EPO gene expression and serum content, improved running and swimming endurance, increased liver and muscle glycogen content, and reduced blood urea nitrogen (BUN) and lactate levels [23]. In humans, the combination significantly improved performance in the 5 km run, 30 m sprint, countermovement jump, and maximal oxygen uptake tests in trained and untrained subjects [23].

In contrast to the findings above, Walker *et al*. [24] reported no benefit of *R. rosea* supplementation following four days of consuming 1500 mg/day in a group of 12 resistance-trained males on an incremental time-to-exhaustion test or RPE during wrist flexion exercise in the non-dominant arm. In a crossover study of caffeine (3 mg/kg), *R. rosea* (3 mg/kg), caffeine (3 mg/kg) + *R. rosea* (3 mg/kg), and placebo; Duncan *et al*. [25] found beneficial effects of caffeine ingestion on 5 km time trial performance; however, *R. rosea* alone did not result in any benefit. Further, *R. rosea* in conjunction with caffeine did not result in greater time trial performance compared with caffeine alone, thus, in context with the results from Yun *et al*., the effect of caffeine ingestion on performance in combination with *R. rosea* is not well understood. In a study of longer duration, four weeks of daily *R. rosea* supplementation (600 mg; 200 mg 3×/day) resulted in enhanced psychomotor performance, reaction time, and response time; however, no improvements in peak oxygen consumption or heart rate responses were observed [26]. Interestingly, at post testing, the placebo condition experienced a significant decrease in peak power output whereas the *R. rosea* group maintained performance.

*rosea* is purported to reduce stress and improve mood and cognitive function [27]. Mechanistically, *R. Rosea* has been demonstrated to enhance neuropeptide-Y expression in neuroglial cells, act an monoamine oxidase (MAO) A and MAO B inhibitor, regulate genes related to behavior and mood, and maintain hypothalamic – pituitary – adrenal (HPA) axis homeostasis [27]. Specifically, salidroside has been shown to enhance neurotransmitter homeostasis by increasing the permeability of the blood–brain barrier to dopamine and serotonin precursors leading to increased concentrations of dopamine and serotonin [28]. Further, salidroside may increase noraderenaline in the brainstem and pre-frontal cortex and promote the proliferation and differentiation of neural stem cells in the hippocampus [28]. Similarly, physical exercise can have numerous psychological effects such as reducing stress level and improving mood and cognitive function [29,30]; thus, a combination of exercise and *R. rosea* or salidroside ingestion may produce additive effects. For example, mood state has been shown to be improved post-exercise with *R. rosea* supplementation to a greater extent than exercise alone [31]. Some previous studies without exercise intervention have demonstrated a positive impact of *R. rosea* on depressive mood and mental performance. Darbinyan, *et al*. [32] reported six weeks of *R. rosea* extract supplementation to be capable of producing anti-depressive effects in people with mild-to-moderate depression. In participants experiencing stress-related fatigue [33], 28 days of *R. rosea* extract supplementation resulted in decreased measures of burnout and greater improvement in psychomotor performance when compared to placebo. However, caution regarding the evidence of *R. rosea* as a mood enhancer is warranted as a systematic review of *R. rosea* indicated that while evidence for improved mood and mental performance resulting from *R. rosea* supplementation exists, the overall evidence for efficacy is inconclusive [34].

*rosea* supplementation has also been studied in the context of oxidative stress, inflammation, and muscle damage resulting from exercise with mixed results in humans. Thirty days of *R. rosea* supplementation in exercise naïve young adults attenuated C-reactive protein (CRP) and creatine kinase (CK) response to exhaustive exercise compared with placebo [35]. Conversely, *R. rosea* supplementation did not improve recovery following a marathon as measured by vertical jump performance and delayed-onset muscle soreness, nor did supplementation reduce circulating inflammatory markers or muscle damage markers [36]. Likewise, inflammation markers resulting from a three-day exercise period failed to be reduced by *R. rosea* supplementation; however, a non-significant trend toward decreased CK was observed [37].

As noted, studies of salidroside supplementation in humans have been restricted to the use of harvested *R. rosea* extracted to access bioactive compounds typically standardized to 1% salidroside [18]. Thus, salidroside concentration in standardized *R. rosea* extracts for human consumption is fairly limited. In order to induce the beneficial effects observed from *in vitro* and *in vivo* animal studies, higher dosages of salidroside intake may be necessary. Due to overharvesting, *R. rosea* was already considered as an endangered plant species as early as 2012 [38], and global demand for *R. rosea* is continuing to rise leading to potentially unsustainable harvest of the relatively scarce *R. rosea* plant [39]. Furthermore, extraction and purification from plant material can lead to co-purification of undesirable compounds, including solvents or other potentially harmful plant metabolites [40]. Recently, the biosynthetic route toward salidroside biosynthesis in *R. rosea* was discovered through transcriptomic analysis and functional characterization [41]. Drawing from this discovery, pure, nature-identical salidroside was produced in a microorganism via precision fermentation, and was found to be highly safe and nontoxic in animal toxicity studies [42]. Additionally, no major drug–drug interactions were detected between biosynthetic salidroside and a panel of major drug classes [43]. The recently developed salidroside manufacturing capabilities will thus enable support of detailed and rigorous study of highly safe, salidroside in human participants while simultaneously promoting the conservation of the wild *R. rosea* plant [39,42]. Therefore, the goal of the current exploratory study was to determine the acute and short-term effects of higher-dose, synthetically produced, salidroside supplementation (60 mg per day) on exercise performance (oxygen uptake, time-to-exhaustion, local muscular endurance, erythropoietin), mood state, and markers of inflammation and muscle damage in young, active, healthy adults.

## 2. Materials and methods

### 2.1. Participants

Fifty healthy active young adults (30 M, 20F; 15 M, 10F per group) completed the study. Participants were recruited via word-of-mouth, social media advertisements, and university e-mail announcements. All participants performed aerobic exercise at least once per week (running or jogging) for at least six months prior to the study and had prior treadmill experience. The study protocol was approved by the University of South Alabama Institutional Review Board (#22-131) and was conducted in accordance with the Declaration of Helsinki. The procedures of the study were explained to the participants and written and verbal informed consent was obtained prior to being enrolled in the study. Participants completed a health history questionnaire and physical activity questionnaire to confirm their eligibility for participation.

### 2.2. Experimental approach

**Figure 1.**
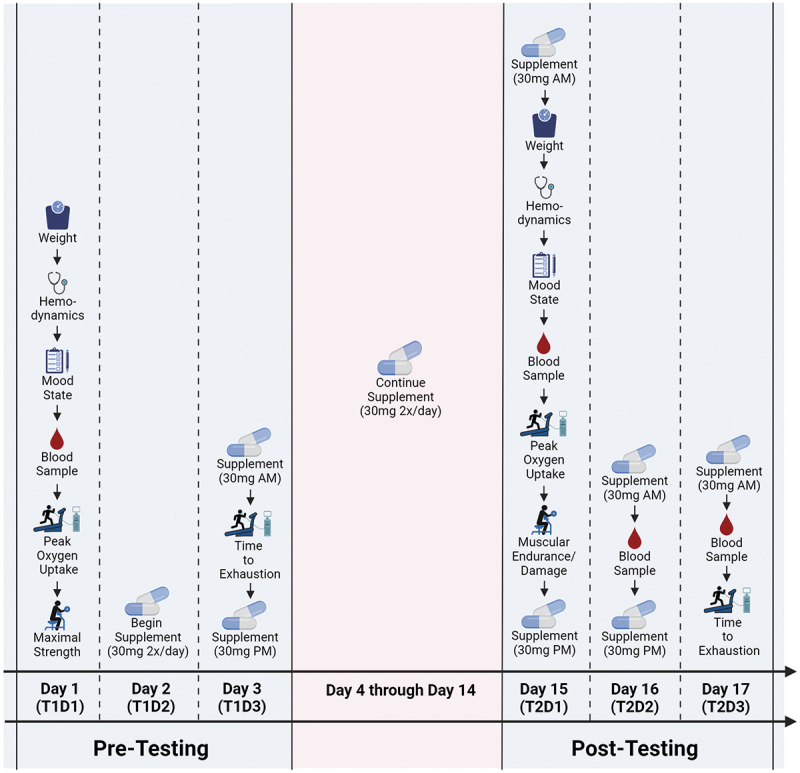
Overview of study design. Created with BioRender.com.

Participants completed an entry session followed by two multi-day testing sessions separated by 14 days (Figure 1). During the entry session, participants were screened for eligibility and explained the study procedures. Verbal and written informed consent were obtained followed by anthropometric measurements and equipment/exercise familiarization. Qualifying and willing participants were scheduled for their testing sessions. Participants were instructed to keep their normal dietary habits except for the consumption of the prescribed supplement and pre-testing instructions. For all testing session days, participants arrived to the lab first thing in the morning having avoided food and drink (except water) for 8–12 hours prior, and to avoid alcohol intake for at least 48 hours prior. Participants were instructed to refrain from caffeine, or other stimulants, the morning of their visit and to refrain from non-study related exercise for 72 hours before each testing time point. Outside of the restricted exercise time frame, participants were instructed to maintain their normal exercise habits.

Upon arrival for the first test visit (T1D1), body mass was assessed followed by relaxation in a chair for ten minutes after which hemodynamics and mood state were assessed. After, a blood sample was obtained and the participants performed a peak oxygen assessment on a treadmill. After the peak oxygen uptake assessment, the participants rested for 20 minutes and a maximal strength test for the elbow flexors was performed utilizing a biceps curl machine which concluded T1D1. The participants began ingesting their assigned supplement the following day (i.e. T1D2). On day three (T1D3), the participants returned for a second laboratory visit to perform a time-to-exhaustion test on a treadmill having consumed their assigned supplement approximately 30 minutes before their scheduled visit. After the conclusion of T1D3, the participants continued consuming their assigned supplement for 14 more days (i.e. throughout the remainder of the study). Participants returned to the lab for visit one post-testing (T2D1) 14 days after T1D1 (i.e. two weeks following their initial laboratory visit).

T2D1 proceeded exactly as T1D1 except participants performed a local muscular endurance and damage protocol for the elbow flexors in lieu of the maximal strength test. In line with T1D1, participants consumed their assigned supplement 30 min prior to their scheduled time for each post-testing laboratory visit. Participants returned to the laboratory 24 hours after their muscle endurance/damage protocol for a blood draw (T2D2). Twenty-four hours after T2D2 (T2D3), participants returned for their final visit where they provided a blood sample followed by a time-to-exhaustion test identical to the one performed at T1D3. After, the participants completed an adverse events questionnaire and their participation in the study was concluded.

### 2.3. Testing protocols

#### 2.3.1. Anthropometric and hemodynamic assessments

Total body mass (kg) was determined by using a calibrated digital scale (Tanita Corp., TBF-400, Arlington Heights, IL). Height (cm) was determined using a stadiometer with a precision of 0.1 cm using standard procedures (Detecto, PHR, Webb City, MO). Blood pressure and heart rate were assessed in a seated position after a ten-minute rest period with an automated brachial blood pressure cuff using standard procedures (Omron Corp., Omron 10, Kyoto, Japan).

#### 2.3.2. Mood state

Mood state was assessed at T1D1 and T2D1 using the Profile of Mood States (POMS) instrument [44]. The POMS instrument is a 65-item questionnaire that assesses seven mood domains (fatigue-inertia, anger-hostility, vigor-activity, confusion-bewilderment, depression-dejection, tension-anxiety and friendliness) as well as total mood disturbance. Participants completed the POMS in an electronic format on their personal electronic device in a distraction-free environment.

#### 2.3.3. Peak oxygen uptake assessment

Participants performed a graded exercise test on a treadmill (Trackmaster Treadmills, TMX428 CP, Newton, KS) at T1D1 and T2D1 to determine their peak oxygen consumption (VO_2_ peak). Gas exchange during the test was measured breath-by-breath by having the participant wear a facemask (Hans Rudolph, Inc., 7450 V2 Series, Shawnee, KS) connected to a metabolic cart system (COSMED, Quark CPET 2.1, Rome, Italy) and the highest level of VO_2_ obtained over a 15-second average was defined as the VO_2_ peak. During the test, a heart rate monitor strap (PowrLabs, Chest Heart Rate Monitor, Park City, UT) was worn across the chest along with a custom-made overhead safety harness. After baseline measurements of gas exchange and heart rate were established, participants warmed-up for six minutes on the treadmill: two minutes at 5.6 km/h and 1% grade, two minutes at 5.6 km/h and 2% grade, and then two minutes at 8.0 km/h and 2% incline grade. After the warm-up, speed was increased to 10.5 km/h at 2% incline grade. The percent grade incline increased 2% every 90 seconds until the participants signaled they could no longer continue at which point the test was ended and a cool-down regimen was performed. Peak heart rate and peak respiratory exchange ratio were calculated using the highest 15-second average for each and recorded at the end of the test.

#### 2.3.4. Time-to-exhaustion protocol

**Figure 2.**
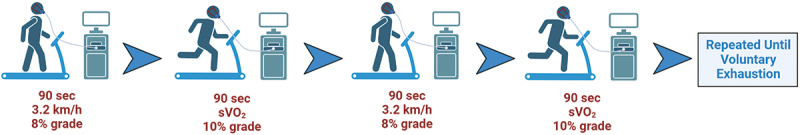
Overview of time-to-exhaustion test protocol. sVO2 = speed associated with the participant’s VO2 peak; created with BioRender.com.

Participants performed a time-to-exhaustion (TTE) test on a treadmill at T1D3 and T2D3 using a high-intensity intermittent exercise (HIIE) protocol (Figure 2). Utilizing the same setup as VO_2_ peak testing (see above), participants wore a facemask connected to a metabolic cart system to analyze oxygen uptake along with a heart rate monitor and custom-made overhead safety harness throughout the duration of the test. The TTE test consisted of 1.5-minute work bouts at the speed associated with their VO_2_ peak (sVO2 peak) at 10% grade, calculated using American College of Sports Medicine (ACSM) metabolic equations [45], followed by active recovery for 1.5 minute at 3.2 km/h at 8% grade until voluntary exhaustion. Before starting the protocol, resting HR and VO_2_ were obtained followed by a three-minute warm-up at 3.2 km/h at 8% grade. The average oxygen uptake for each 90 second work bout was determined and divided by the estimated oxygen uptake requirement, according to the ACSM metabolic equations [45], resulting in a calculated percent predicted oxygen uptake for each 90 sec work bout [46]. Participants were instructed to complete as many work intervals as possible and the total number of intervals completed was recorded for each participant. Participants were not informed of how many intervals they had completed if they failed to maintain a personal count in order to avoid introducing bias. Consistent verbal encouragement was provided by the research team throughout the TTE. If a participant stopped in the middle of a work interval, the fraction of the interval completed was included in the total number of intervals. Blood lactate was measured from the fingertips at rest, immediately after exercise, and three, five, seven, and ten minutes after exercise using a handheld device (Lactate Pro 2 LT-1730, Arkray Inc., Japan). The peak blood lactate measurement post-exercise was recorded so that baseline and peak blood lactate measurements were obtained, and delta lactate levels (Δ lactate) calculated by subtracting baseline lactate from peak post-exercise lactate at each time point.

#### 2.3.5. Maximal strength assessment of the elbow flexors

Maximal strength of the elbow flexors was assessed on a biceps curl machine (Maxicam, Arm Curl Selectorized Strength Machine, Long Beach, CA). The participant was positioned on the machine and settings modified for comfort. Once set, a conservative amount of weight was added to the machine and the participant performed six to ten warm-up repetitions followed by a one-minute rest. Conservatively, more weight was added based on the participant’s subjective assessment of exertion during the first set. For the second set, the participant performed three to five repetitions. After a one-minute rest, more weight was added and one repetition was performed. The participant continued to do sets of one repetition with increasing weight with two minutes rest between attempts until they could no longer successfully complete a repetition. The highest amount of weight used for a successful repetition was considered their maximal strength (1-RM). The maximal strength assessment was only performed at T1D1.

#### 2.3.6. Local muscular endurance assessment

The same biceps curl machine used for the maximal strength test was utilized for the local muscular endurance test. The local muscular endurance test also functioned as a protocol to induce muscle damage. Participants performed a warm-up at 50% of their measured 1-RM for two sets of eight repetitions. After the warm-up sets, participants completed eight work sets for as many repetitions as possible during each set. Two minutes of rest was allowed between each of the eight work sets. The load of the work sets was 90% 1-RM for the first three sets, 70% 1-RM for the next three sets, and 50% 1-RM for the final two sets. The local muscular endurance test was only performed at T2D1 and consistent verbal encouragement was provided by the research team throughout the test.

#### 2.3.7. Venous blood sampling

Venous blood samples were obtained via venipuncture from the antecubital vein into blood collection tubes using a standard Vacutainer apparatus. Standard phlebotomy procedures were employed using a 21-gauge needle or smaller. Briefly, the puncture site was cleaned with 70% alcohol pad and allowed to dry before proceeding. After, a tourniquet was applied 3 to 4 inches above the collection site. Approximately 30 mL of blood was obtained at each sample time point for a total of 120 mL for the study over a two-week period. A small amount of blood from the samples at T1D1 and T2D1 were outsourced to LabCorp Inc., Birmingham, AL for complete blood count (CBC) and comprehensive metabolic panel (CMP) analyses. The remaining blood (serum and plasma) was immediately processed and stored at −80°C until analyses. From the stored blood, erythropoietin (EPO) was determined from plasma samples collected at T1D1 and T2D1. Creatine kinase skeletal muscle-specific isozyme (CK-MM), and highly sensitive C-reactive protein (hs-CRP) were measured from plasma samples collected at T2D1, T2D2, and T2D3. Myoglobin (MYO) levels were measured from serum samples obtained at T2D1, T2D2, and T2D3. The time points of T2D1, T2D2, and T2D3 corresponded with pre-exercise (Pre-Ex), 24-hour post-exercise (24PoEX), and 48-hour post-exercise (48PoEx) relative to the peak oxygen uptake and local muscular endurance (i.e. muscle damage protocol) tests.

#### 2.3.8. Blood marker analyses

Analyses of EPO (Human Erythropoietin/EPO ELISA Kit PicoKine®, Boster Biological Technology, Pleasanton CA, USA, Catalog # EK0332), MYO (Myoglobin ELISA, DRG International, Springfield NJ, USA, Catalog # EIA3955), CK-MM (Human Creatine Kinase MM isoenzyme ELISA Kit, AFG Bioscience, Northbrook IL, USA, Catalog # EK711916), and hs-CRP (C-Reactive Protein HS ELISA, DRG International, Springfield NJ, USA, Catalog # EIA3954) were performed in duplicate using a plate-based colorimetric measurement (SpectraMax 384 Plus, and SoftMax Pro Software, Molecular Devices, San Jose, CA) according to manufacturer specifications. The intra-assay coefficient of variance (CV) values for EPO, MYO, CK-MM, and hs-CRP were 15.7%, 11.3%, 11.6%, and 4.6%, respectively.

### 2.4. Supplementation protocol

The test product used in this study was salidroside as LANDKIND® Pure Salidroside. LANDKIND® is a registered trademark of Lesaffre & Cie (France). Participants were instructed to consume 60 mg of salidroside (SAL; *n* = 25, 15 M, 10F each group) or rice flour placebo (PLA) daily divided into two doses of a single 30 mg capsule each morning and afternoon. The dosage amount was determined based on unpublished pilot data obtained prior to starting the study. During pilot testing (University of South Alabama Institutional Review Board #22-044), five participants consumed single 30 mg, 60 mg, 120 mg, and 180 mg doses of salidroside and placebo (rice flour) on separate days in a randomized crossover fashion. No adverse events were observed or reported for any condition. Since no acute adverse events were observed up to 180 mg, the dose of 60 mg (30 mg 2× daily) was chosen for ecological validity reasons as this was consistent with product labeling. Participants were instructed to consume their first dose upon waking and to consume their second dose at least six hours later. Salidroside and placebo were supplied by LandKind, Inc., a subsidiary of DoubleRainbow Biosciences (Lexington MA, USA). Participants were block-randomized to their supplement group based on peak oxygen uptake and biological sex using a random number generator (www.random.org). Participants were randomized after their first peak oxygen uptake assessment. If their VO_2_ peak was within 5 ml/kg/min of O_2_ to a participant of the same sex, they were automatically placed in the opposite group. If no prior unmatched participant was available, they were randomized to a group using the random number generator. Supplementation began the morning of T1D2 and continued through the remainder of the study (31 total doses with the last dose ingested on the morning of T2D3). On testing days, participants were instructed to consume their morning dose 30 minutes before their scheduled testing time. Thus, for testing on T1D3 participants had consumed three doses of their assigned supplement (two on T1D2 and one 30 min before their scheduled testing on T1D3) in order to allow for study of acute consumption on TTE test performance. Participants were instructed to log the consumption of each dose on a sheet provided to them. The supplement was double-blinded to the participants and the research team by a researcher uninvolved with data collection (R.J.C.) with unblinding occurring after analyses were complete.

### 2.5. Self-reported adverse events

Participants self-reported the presence of adverse events for headache, fever, vomiting, lethargy/fatigue, loss of appetite, insomnia, depression, anxiety, skin rash, diarrhea, dry mouth, dizziness, shortness of breath, blurred vision, fast heart rate/palpitations, nervousness, upset stomach or nausea, and drowsiness using a visual analog scale at the end of study participation.

### 2.6. Statistical analyses

Sample size was calculated considering the difference in peak oxygen consumption as the main variable of the study. Using G*Power version 3.1.9.6 [47], an *a priori* power analysis was performed for sample size determination using a significance criterion of *α* = 0.05, power of 0.80, and a medium effect size of 0.5. The minimum total sample size needed with these criteria was 34 participants for a repeated measures test examining within-between interactions. Thus, the obtained sample size of *N* = 50 was adequate to test the study hypothesis. Data for each group at each time point were checked for normality of distribution using the Kolmogorov–Smirnov test with Lilliefors correction. Of the data checked, only MYO, CK-MM, hs-CRP and self-reported adverse events were determined to have non-normal distribution requiring non-parametric testing. For all data, results are presented as mean ± standard deviation (SD).

For POMS, peak oxygen consumption, CBC, CMP, EPO, blood pressure, number of TTE intervals and TTE Δ lactate data, separate mixed-model 2 × 2analysis of variance (ANOVA) were utilized to determine the effect of each supplement (between-factor) over time (within-factor). For each variable, main effects and within group simple effects were analyzed using paired samples *t* tests for time comparisons and independent samples *t* tests for group comparisons with Bonferroni’s correction applied. For analyses of the local muscular endurance test, number of repetitions for each set at each load intensity were summed, and independent *t* tests for total number of repetitions at 90%, 70%, 50%, and total overall repetitions were performed to compare between groups. For MYO, CK-MM, and hs-CRP, related-samples Friedman’s two-way ANOVA by ranks was utilized to compare time points within groups. Additionally, independent-samples Mann-Whitney U tests were employed to determine differences between groups at each time point for these blood markers and self-reported adverse events.

For statistical analyses of oxygen consumption kinetics during the TTE tests, average oxygen consumption during each work interval was divided by the predicted oxygen consumption required to perform the task resulting in a percentage value [45]. Since every participant was able to complete at least three work intervals, the first three work intervals were calculated individually. Beyond that, participants completing 5 or more work intervals had their remaining work intervals split in half and averaged as middle work intervals and final work intervals (FI) resulting in five values for each participant for each TTE test: first work interval (Interval 1), second work interval (Interval 2), third work interval (Interval 3), Middle work Intervals (MI), and Final work Intervals (FI). If the remaining work intervals were odd in number, the additional work interval was grouped with MI. First, the percent of predicted oxygen uptake obtained for each work interval grouping was compared between groups at T1D3 and T2D3 using separate 2 × 5(group x interval) mixed-effect models using the restricted maximum likelihood method because of the missing values associated with those that only completed three work intervals (i.e. no MI or FI data). Bonferroni’s correction was applied to between group analyses. Next, similar to before, the percent of predicted oxygen uptake obtained for each work interval group was compared within groups between T1D3 and T2D3 using a 2 × 5(session x interval) within-within model with Bonferroni’s correction applied. In addition, the oxygen uptake percentage for each work interval grouping and session (T1D3 and T2D3) was collapsed to compare overall percent oxygen uptake between groups using independent samples *t* test. Lastly, percent oxygen uptake was combined by session (T1D3 and T2D3) and group (PLA and SAL) to determine overall oxygen uptake differences between intervals using univariate ANOVA with Bonferroni’s correction applied.

Effect sizes for significant main effects and significant simple main effects were calculated as Cohen’s *d* using Microsoft Excel ® 2019 (Microsoft Corporation, Redmond WA, USA). The values of Cohen’s *d* for small, medium and large effects were 0.2, 0.5 and 0.8, respectively. Statistical analyses were performed using SPSS Statistics 28.0 (IBM Corp.; Armonk NY, USA) and GraphPad Prism 10.2.2 (GraphPad Software, LLC.; Boston, MA, USA). An *a priori* probability level of ≤0.05 was adopted.

## 3. Results

### 3.1. Baseline participant data and CONSORT flow diagram

**Figure 3.**
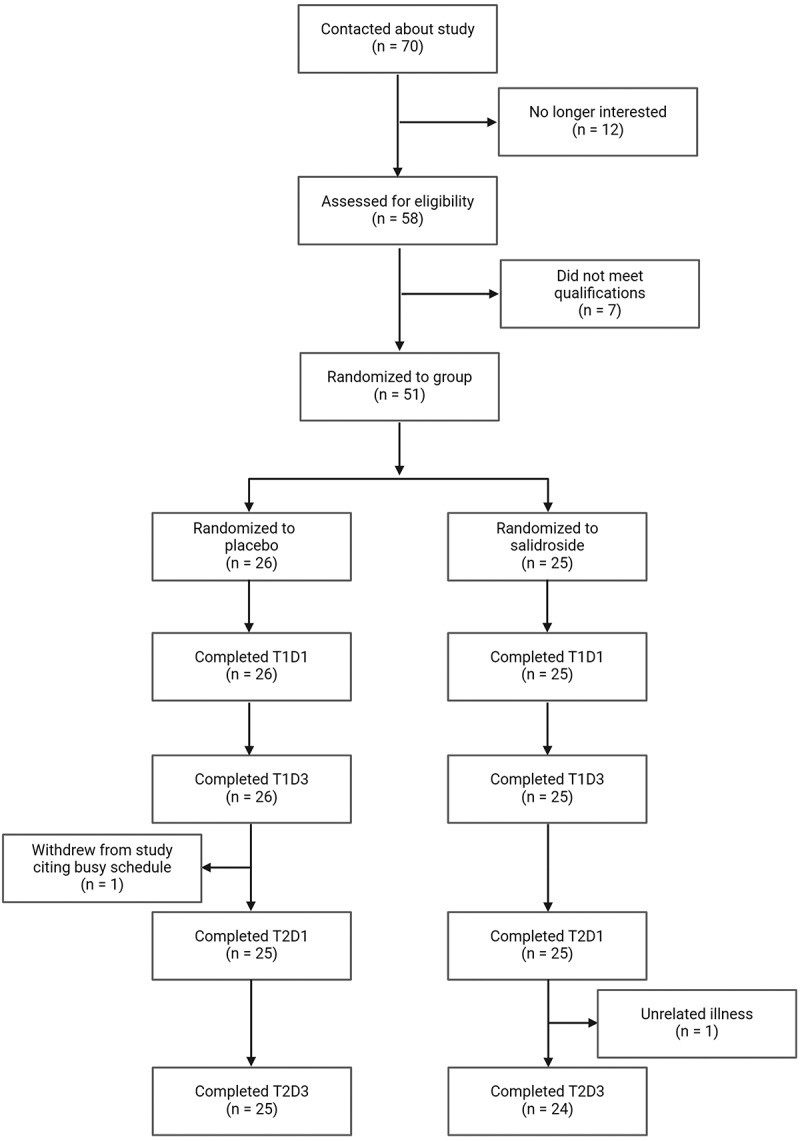
CONSORT flow diagram. Created with BioRender.com.

Fifty healthy active young adults completed the study. One participant for the SAL group did not complete T2D3 due to unrelated illness. A CONSORT flow diagram is depicted in Figure 3. Participants for both groups reported supplement ingestion compliance >99%. Baseline characteristics of participants are described in Table 1.

**Table 1.** Baseline characteristics of participants (*M ± SD*).

Age (years)	21.1 ± 3.9
Height (cm)	173.0 ± 7.6
Body Mass (kg)	74.4 ± 13.4
Relative VO_2peak_ (ml•kg^−1^•min^−1^)	44.8 ± 5.4
Maximal Strength (kg)	48.2 ± 19.1

*M ± SD* denotes mean ± standard deviation. VO_2peak_ denotes peak rate of oxygen consumption.

### 3.2. Time-to-exhaustion

**Figure 4.**
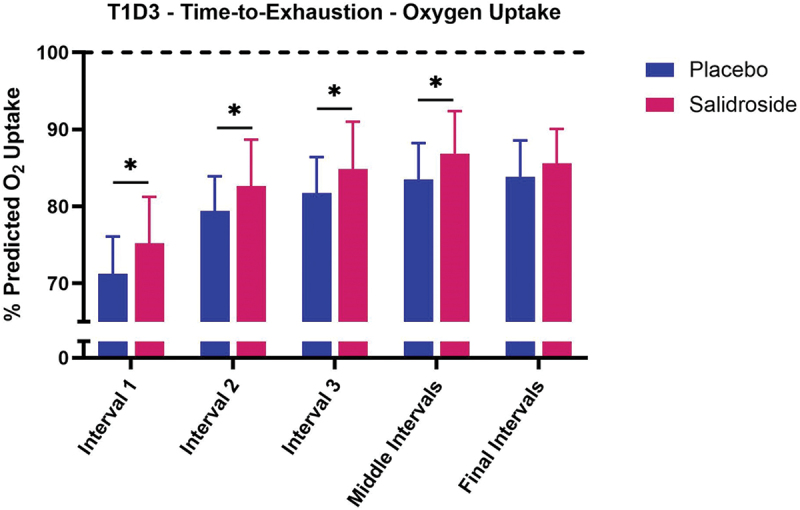
Effect of salidroside on oxygen uptake during time-to-exhaustion test at T1D3. The bars represent the mean percentage of predicted oxygen uptake (% predicted O₂ uptake) across work intervals during the time-to-exhaustion test for salidroside (red bars) and placebo (blue bars). Significant differences between the placebo and salidroside groups are indicated by asterisks (*), with salidroside showing a higher percentage of predicted O₂ uptake at interval 1, interval 2, interval 3, and middle intervals. No significant difference is observed during final intervals. Error bars represent standard deviations.

**Figure 5.**
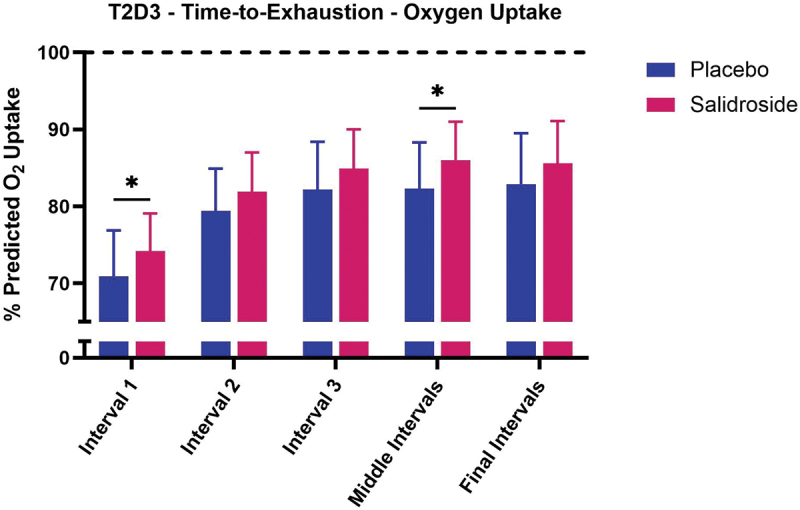
Effect of salidroside on oxygen uptake during time-to-exhaustion test at T2D3. The bars represent the mean percentage of predicted oxygen uptake (% predicted O₂ uptake) across work intervals during the time-to-exhaustion test for salidroside (red bars) and placebo (blue bars). Significant differences between the placebo and salidroside groups are indicated by asterisks (*), with salidroside showing a higher percentage of predicted O₂ uptake at interval 1 and middle intervals. No significant differences are observed during interval 2, interval 3, and final intervals. Error bars represent standard deviations.

**Figure 6.**
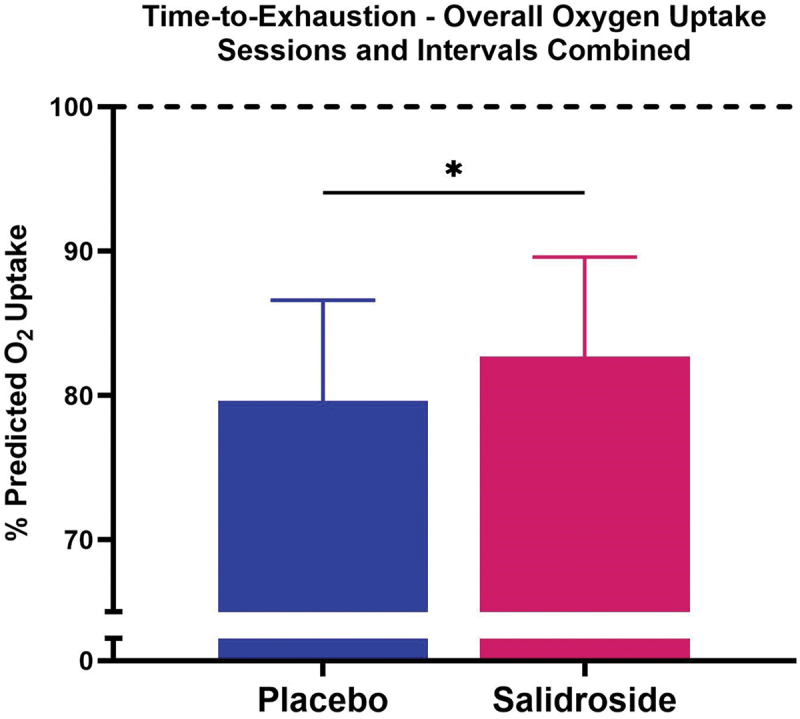
Overall effect of salidroside on percent predicted oxygen uptake during time-to-exhaustion tests. The bars represent the mean overall percentage of predicted oxygen uptake during the time-to-exhaustion tests, combining both sessions (T1D3 and T2D3) and all work intervals for both placebo (blue bar) and salidroside (red bar) groups. The salidroside group shows significantly higher overall oxygen uptake compared to the placebo group, as indicated by the asterisk (*). Error bars represent standard deviations.

**Figure 7.**
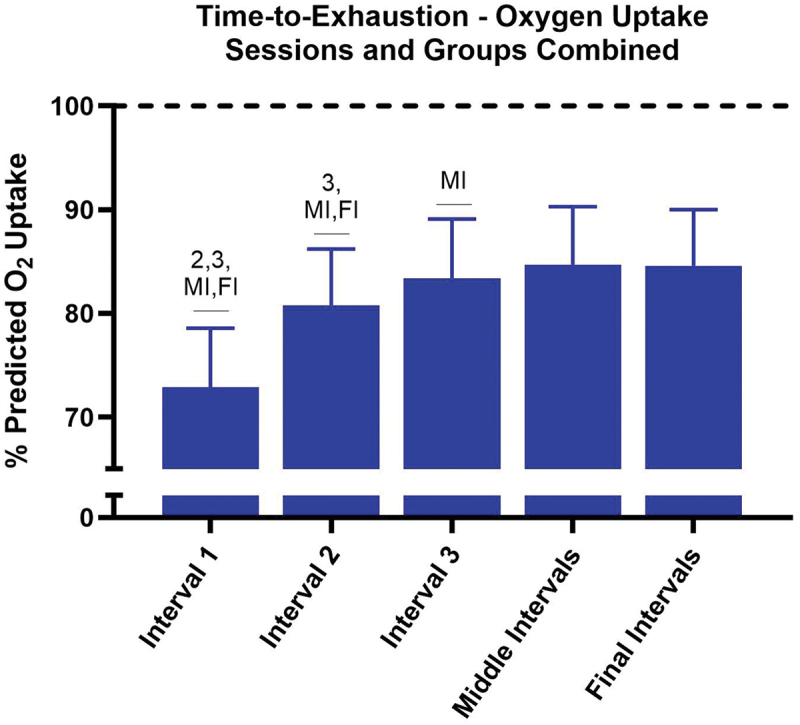
Percent predicted oxygen uptake for each interval grouping (sessions and groups collapsed) during time-to-exhaustion tests. The bars represent the mean percentage predicted oxygen uptake for each interval grouping during the time-to-exhaustion tests, combining both sessions and groups. Percentage predicted oxygen uptake for interval 1 is significantly lower than interval 2, interval 3, middle intervals (MI), and final intervals (FI). Interval 2 is significantly lower than interval 3, MI, and FI. Interval 3 is significantly lower than MI. Error bars represent standard deviations.

No significant interaction between group and interval existed for percent predicted oxygen uptake at T1D3 (*p* = 0.28) or T2D3 (*p* = 0.75). For T1D3 (Figure 4), SAL demonstrated higher percent predicted oxygen uptake for work Interval 1 (*p* < 0.01; Cohen’s *d* = 0.73), work Interval 2 (*p* = 0.03; Cohen’s *d* = 0.62), work Interval 3 (*p* = 0.03; Cohen’s *d* = 0.57), and Middle work Intervals (*p* = 0.03; Cohen’s *d* = 0.66) compared with PLA. No difference was observed between groups for Final work Intervals (*p* = 0.20). For T2D3 (Figure 5), SAL demonstrated higher percent predicted oxygen uptake for work Interval 1 (*p* = 0.04; Cohen’s *d* = 0.60) and Middle work Intervals (*p* = 0.04; Cohen’s *d* = 0.68) compared with PLA. No difference was observed between groups for work Interval 2 (*p* = 0.10), work Interval 3 (*p* = 0.11), or Final work Intervals (*p* = 0.16). For both PLA and SAL, no differences were observed between sessions T1D3 and T2D3 for percent predicted oxygen uptake during each work interval grouping (all *p* > 0.05). When collapsing sessions and work intervals, overall percent oxygen uptake was greater for SAL compared with PLA (*p* < 0.01; Cohen’s *d* = 0.45; 82.7 ± 6.9% vs. 79.6 ± 7.0%, Figure 6). When data were collapsed for group and session (Figure 7), univariate analysis revealed significant differences in percent predicted oxygen uptake between work intervals with work Interval 1 significantly lower (all *p* < 0.01) than work Interval 2 (Cohen’s *d* = 1.42), work Interval 3 (Cohen’s *d* = 1.84), Middle work Intervals (Cohen’s *d* = 2.09), and Final work Intervals (Cohen’s *d* = 2.11). Work Interval 2 was significantly lower (all *p* < 0.01) than work Interval 3 (Cohen’s *d* = 0.47), Middle work Intervals (Cohen’s *d* = 0.71), and Final work Intervals (Cohen’s *d* = 0.70). Work Interval 3 was significantly lower than Middle work Intervals (*p* < 0.01; Cohen’s *d* = 0.23) with no difference from Final work Intervals (*p* = 0.08). No significant difference was observed between Middle work Intervals and Final work Intervals (*p* > 0.99).

**Figure 8.**
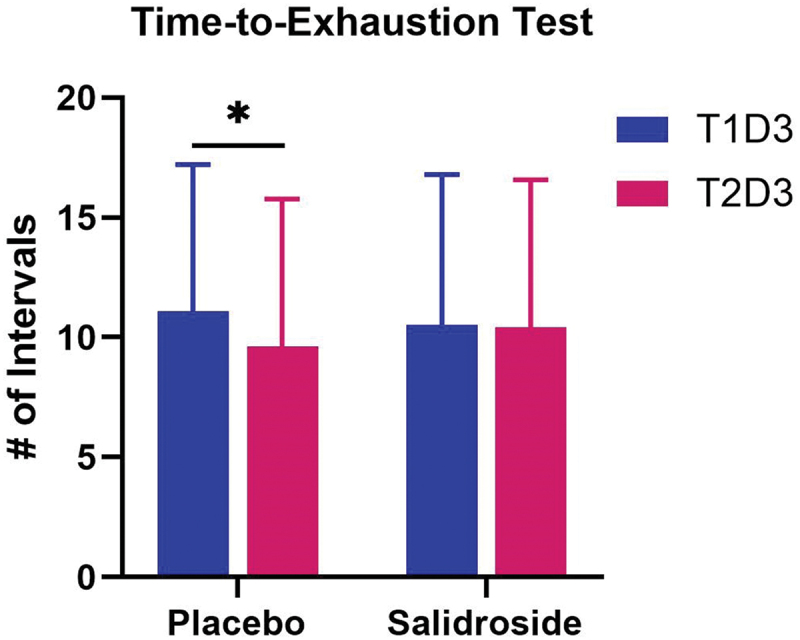
Number of intervals completed by each group during time-to-exhaustion tests at each session. The bars represent the mean number of intervals completed during the time-to-exhaustion tests at T1D3 (blue bars) and T2D3 (red bars) for the placebo and salidroside groups. The placebo group performed significantly fewer intervals at T2D3 compared with T1D3, as indicated by the asterisk (*). Error bars represent standard deviations.

No significant interaction effect (*p* = 0.18) was observed for number of work intervals completed during the TTE test. Within group analyses revealed significantly fewer work intervals performed at T2D3 compared with T1D3 for the PLA group (*p* = 0.03; Cohen’s *d* = 0.39), whereas number of work intervals performed at T1D3 and T2D3 for SAL were almost identical (Figure 8). No significant interaction effect (*p* = 0.81) was observed for Δ lactate values resulting from the time-to-exhaustion tests. A significant main effect of time was observed (*p* = 0.02) where T2D3 Δ lactate values (11.6 ± 4.1 mmol/L) were significantly lower than T1D3 Δ lactate values (13.0 ± 4.5 mmol/L).

### 3.3. Profile of mood state

**Figure 9.**
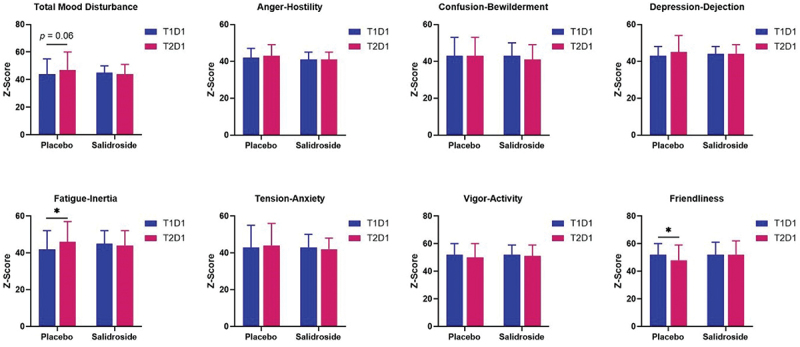
Profile of mood states output for each dimension by group and time point. The bars represent the mean Z-score for each profile of mood states (POMS) dimension at T1D1 (blue bars) and T2D1 (red bars) for placebo and salidroside groups. The placebo group shows significantly higher fatigue-inertia and significantly lower friendliness at T2D1 compared with T1D1, as indicated by the asterisks (*). A trend for greater total mood disturbance at T2D1 compared with T1D1 was observed for the placebo group (*p* = 0.06). Error bars represent standard deviations.

Trends for a significant interaction effect were observed for friendliness (*p* = 0.07) and total mood disturbance (*p* = 0.08). Within-group analysis revealed a significant decrease of friendliness (*p* < 0.01; Cohen’s *d* = 0.49) and a trend for an increase in total mood disturbance (*p* = 0.06) from T1D1 to T2D1 in the PLA group, whereas no significant difference was observed for either for SAL (*p* > 0.05 for both). A significant interaction effect (*p* = 0.02) was observed for fatigue-inertia. Analysis of simple main effects revealed no significant difference between groups at T1D1 (*p* = 0.24) or T2D1 (*p* = 0.33). No significant difference was observed between T1D1 and T2D1 for SAL (*p* = 0.62), whereas a significant increase was observed for PLA (*p* < 0.01; Cohen’s *d* = 0.48) from T1D1 to T2D1. Results revealed no significant effects for anger-hostility, confusion-bewilderment, depression-dejection, tension-anxiety, or vigor-activity (all *p* > 0.05). Results for POMS are shown in Figure 9.

### 3.4. Peak oxygen consumption

**Figure 10.**
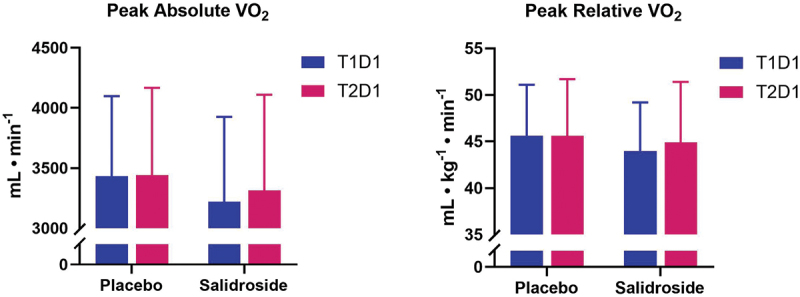
Peak absolute (A) and relative oxygen consumption (B) by group and time point. The bars represent mean peak absolute oxygen consumption (VO₂; panel A) and peak relative VO₂ (panel B) for salidroside and placebo at T1D1 (blue bars) and T2D1 (red bars). No significant differences observed. Error bars represent standard deviations.

No significant differences were observed for absolute or relative peak oxygen consumption (all *p* > 0.05; Figure 10). No significant interaction effect (*p* = 0.32) or main group effect (*p* = 0.29) was observed for maximal heart rate; however, a significant main effect for time was observed (*p* < 0.01) with maximal heart rate significantly lower at T2D1 (190 ± 10 bpm) compared with T1D1 (193 ± 9 bpm). No significant interaction effect (*p* = 0.97) or main group effect (*p* = 0.20) was observed for maximal respiratory exchange ratio. Similar to maximal heart rate, a significant main effect for time for respiratory exchange ratio was observed (*p* = 0.02) with values significantly lower at T2D1 (1.15 ± 0.09) compared with T1D1 (1.18 ± 0.11).

### 3.5. Local muscular endurance

**Figure 11.**
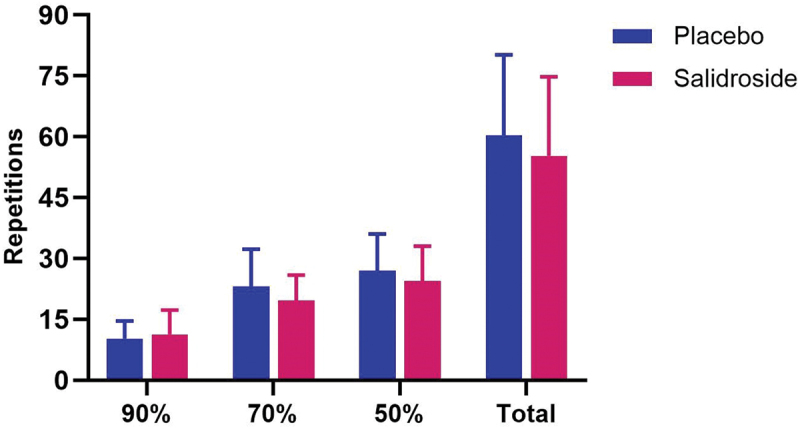
Total repetitions and repetitions performed at each load intensity. The bars represent the mean number of total repetitions performed and number of repetitions performed at each intensity of one-repetition maximum (1-RM) for both placebo (blue bars) and salidroside (red bars) groups. No significant differences in repetitions performed between groups were observed. Error bars represent standard deviations.

No significant differences were observed between groups for number of repetitions performed at 90% of 1-RM (*p* = 0.49), 70% of 1-RM (*p* = 0.13), or 50% of 1-RM (*p* = 0.30). Additionally, no significant difference was observed between groups for total number of repetitions performed (*p* = 0.37; Figure 11).

### 3.6. Blood markers

#### 3.6.1. Comprehensive metabolic panel and complete blood count

Mean values for all variables stayed within normal laboratory range. No significant differences were observed for glucose, creatinine, estimated glomerular filtration rate (eGFR), sodium, potassium, chloride, carbon dioxide, albumin/globulin (A/G) ratio, bilirubin, alkaline phosphatase (ALP), aspartate aminotransferase (AST), alanine transaminase (ALT), white blood cell count (WBC), mean corpuscular volume (MCV), mean corpuscular hemoglobin (MCH), mean corpuscular hemoglobin concentration (MCHC), red cell distribution width (RDW), or platelets (*p* > 0.05 for all).

No significant interaction or main effect of group were observed (all *p* > 0.05); however, a significant main effect of time was observed for calcium (T1D1 > T2D1; *p =* 0.01), albumin (T1D1 > T2D1; *p* = 0.01), globulin (T1D1 > T2D1; *p* = 0.04), red blood cell count (T1D1 > T2D1; *p* = 0.02), hemoglobin (T1D1 > T2D1; *p* < 0.01), and hematocrit (T1D1 > T2D1; *p* = 0.02). A significant interaction effect was observed for blood urea nitrogen (BUN; *p* = 0.02). Blood urea nitrogen significantly increased from T1D1 to T2D1 for PLA (*p* < 0.01). Further, BUN was significantly higher for PLA than SAL at T2D1 (*p* = 0.03). A significant interaction effect was observed for protein (*p* = 0.05). Protein significantly decreased from T1D1 to T2D1 for SAL (*p* < 0.01) with no change in PLA. No changes resulted in mean values moving out of normal reference range.

#### 3.6.2. Erythropoietin, myoglobin, creatine kinase-mm, highly sensitive C-reactive protein

**Figure 12.**
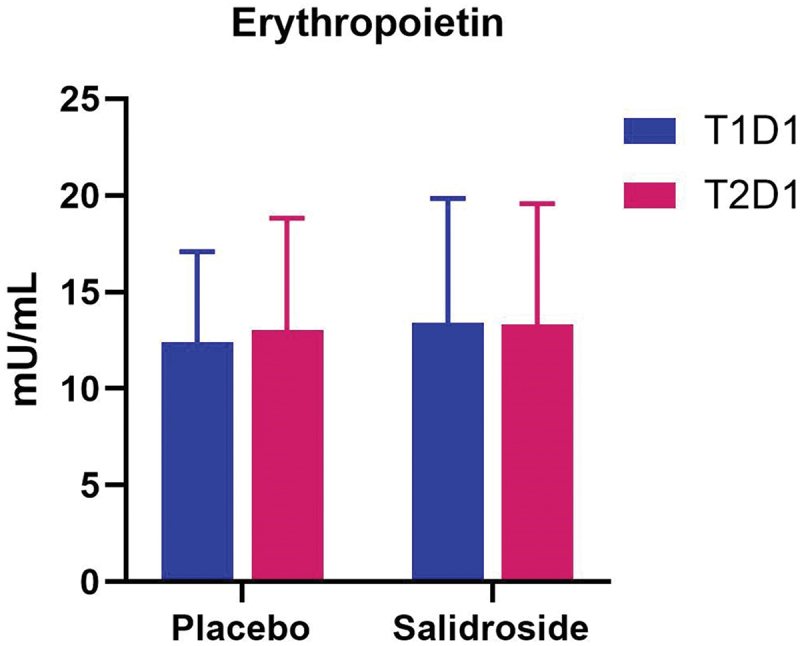
Erythropoietin (EPO) plasma levels by group and time point. The bars represent the mean concentration of plasma erythropoietin (EPO) at T1D1 (blue bars) and at T2D1 (red bars) for both placebo and salidroside groups. No significant differences in EPO were observed. Error bars represent standard deviations.

**Figure 13.**
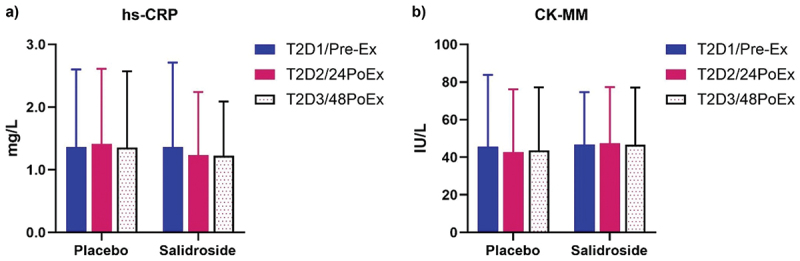
Highly sensitive C-reactive protein (hs-crp, panel A) and muscle-specific creatine kinase (CK-MM, panel B) plasma levels by group and time point. The bars represent the mean concentration of (A) highly sensitive C-reactive protein (hs-crp) and (B) muscle-specific creatine kinase (CK-MM) before exercise at T2D1 (pre-Ex; blue bars), at 24 hours post-exercise on T2D2 (24PoEx; red bars), and at 48 hours post-exercise on T2D3 (48PoEx; dotted bars) for both placebo and salidroside groups. No significant differences in hs-crp or CK-MM levels were observed. Error bars represent standard deviations.

**Figure 14.**
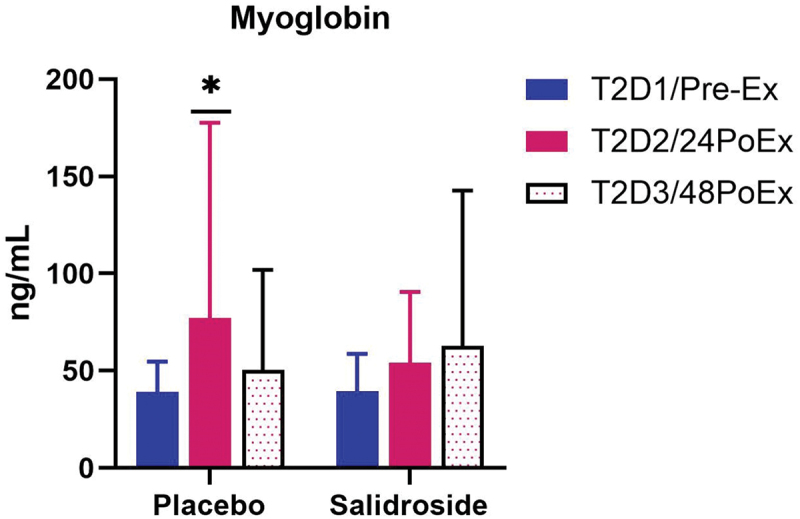
Serum myoglobin (MYO) levels by group and time point. The bars represent the mean concentration of serum myoglobin (MYO) before exercise at T2D1 (pre-Ex; blue bars), at 24 hours post-exercise on T2D2 (24PoEx; red bars), and at 48 hours post-exercise on T2D3 (48PoEx; dotted bars) for both placebo and salidroside groups. MYO was significantly higher at T2D2/24PoEx compared with the other two time points for the placebo group as denoted by the asterisk (*). Error bars represent standard deviations.

No statistically significant changes were observed for EPO (all *p* > 0.05; Figure 12). Due to non-normality of the data, within-group and between-group analyses for hs-CRP, CK-MM, and MYO were performed using related-samples Friedman’s two-way ANOVA by ranks and independent-samples Mann–Whitney U tests, respectively. No within- or between-group differences were observed for hs-CRP or CK-MM (Figure 13; all *p* > 0.05). A significant difference for serum MYO (Figure 14) was observed over time for PLA (*p* < 0.01), but not SAL (*p* = 0.43). Serum MYO was significantly higher at T2D2 (24PoEx) than T2D1 (Pre-Ex) and T2D3 (48PoEx; *p* = 0.02 for both) for PLA with no differences observed for SAL. Mann-Whitney *U* test did not reveal any significant differences between groups at T2D1 (Pre-Ex; *p* = 0.87), T2D2 (24PoEx; *p* = 0.47), or T2D3 (48PoEx; *p* = 0.73).

### 3.7. Self-reported adverse events and blood pressure

Due to non-normality of self-reported adverse event data, groups were compared using independent-samples Mann–Whitney U tests. No significant between group differences were observed for presence of headache, fever, vomiting, lethargy/fatigue, loss of appetite, insomnia, depression, anxiety, skin rash, diarrhea, dizziness, shortness of breath, blurred vision, fast heart rate/palpitations, nervousness, upset stomach or nausea, or drowsiness (all *p* > 0.05) for the duration of the study. The PLA group reported significantly higher levels of dry mouth (*p* = .04) compared with SAL. No significant interaction effect (*p* = 0.06) was observed for systolic blood pressure. Within-group analyses for systolic blood pressure revealed a significant decrease from T1D1 to T2D1 for PLA (*p* = 0.03; Cohen’s *d* = 0.45; T1D1 = 118 ± 11 mmHg, T2D1 = 114 ± 9 mmHg), but not SAL (*p* = 0.70; T1D1 = 115 ± 10 mmHg, T2D1 = 116 ± 9 mmHg). No significant differences for diastolic blood pressure were observed (PLA: T1D1 = 72 ± 6 mmHg, T2D1 = 72 ± 5 mmHg; SAL: T1D1 = 75 ± 9 mmHg, T2D1 = 74 ± 8 mmHg). Overall, salidroside was well tolerated by the participants.

## 4. Discussion

The current study is the first evaluation of supplementation with pure, biosynthetically produced salidroside in humans. As such, the study was designed in an exploratory manner to investigate a wide range of outcomes for which short-term salidroside supplementation may be of benefit as a purported adaptogen with anti-inflammatory, antioxidant, and anti-fatigue properties [7,8,13,48].

### 4.1. Salidroside enhances the rate of oxygen uptake

The ability of acute SAL supplementation to enhance oxygen uptake during exercise was the main finding of the current study. Time-to-exhaustion tests consisting of HIIE on a treadmill were performed on T1D3 and T2D3. Since supplementation started on the morning of T1D2, the first TTE test compared outcomes between acute (two doses the day prior to testing and one dose the morning of testing) PLA and SAL supplementation and the second TTE test compared outcomes between short-term (14 days) PLA and SAL supplementation. Metabolic equations were employed to estimate the treadmill speed associated with participants’ peak oxygen consumption (sVO2 peak) at 10% grade. The average oxygen uptake for each work bout was determined and divided by the expected oxygen requirement resulting in percent oxygen uptake. Within groups, no difference was observed for percent oxygen uptake between T1D3 and T2D3; however, at both time points SAL supplementation resulted in significantly higher percent oxygen uptake compared with placebo. These results suggest as few as three 30 mg doses of salidroside can enhance oxygen uptake. Since an additional two weeks of supplementation did not further enhance oxygen uptake, the effect of SAL supplementation on oxygen uptake during HIIE appears to be the result of acute intake. Potential explanatory mechanisms for augmented oxygen uptake via SAL supplementation exist through its role as an AMPK activator and vasodilator. Evidence suggests AMPK activation may be important for oxygen supply to body tissues through a contribution to control of the hypoxic ventilatory response at the brainstem and ventilation-perfusion matching at the lungs [49]. Additionally, AMPK deficiency has been shown to impair the ventilatory response to hypoxic conditions [50]. Improved ventilatory drive in conjunction with increased vasodilatory response [9], accelerated skeletal muscle substrate mobilization [8], and activation of glycolytic and oxidative enzymes [51] are all outcomes of AMPK activation that may contribute to more efficient oxygen uptake. It is plausible that short-term SAL supplementation may not induce meaningful increases in peak oxygen consumption, but may facilitate better oxygen kinetics resulting in more efficient oxygen utilization during high-intensity efforts as demonstrated by the higher percent of predicted oxygen uptake obtained during TTE tests. Lastly, two weeks of salidroside supplementation did not increase circulating EPO concentration. Despite previous evidence of enhanced erythropoiesis [11], the current study suggests SAL supplementation at the current dosage and intake duration does not result in increased EPO production. Higher doses and/or duration of supplementation may be necessary to observe changes in EPO production.

### 4.2. Salidroside may reduce muscle damage after resistance exercise

Salidroside failed to improve performance during a resistance exercise-based local muscular endurance test of the elbow flexors. Another study reported a significant reduction in repetitions to failure on the bench press exercise following *R. rosea* supplementation, although greater mean concentric velocity was observed [22]. The results of both studies additively suggest no benefit of *R. rosea* or salidroside for resistance exercise volume. Unlike *R. rosea* supplementation, salidroside supplementation in the current study did not significantly reduce the number of repetitions performed suggesting salidroside may not be detrimental to resistance exercise volume capacity. Interestingly, the current study did not observe any changes in hs-CRP or CK-MM resulting from exhaustive exercise (Figures 13 and 14, respectively). However, an increase in serum myoglobin was observed at T2D2 (24PoEx) for the PLA group whereas no increase was observed for the SAL group (Figure 14). Intense exercise can result in structural tearing of skeletal muscle fibers and leakage of muscle proteins into circulation [52]. An animal model of exhaustive swimming combined with salidroside administration found increased antioxidant activity and a reduction of ultrastructural lesions which is consistent with our inability to observe an increase in serum myoglobin for the SAL group [12]. Further research is needed to determine the mechanism behind salidroside’s ability to mitigate muscle damage.

### 4.3. Salidroside may attenuate mood disturbance and prevent decline in high-intensity exercise performance

Mood state was assessed with the POMS instrument on the first day of each time point (T1D1 and T2D1). In comparison to T1D1, the PLA group reported higher levels of fatigue-inertia and lower levels of friendliness at T2D1. The SAL group did not report any changes in mood state. Similarly, the PLA group completed significantly fewer TTE exercise intervals at T2D3 compared with T1D3, whereas the SAL group performed equally as well at both time points (Figure 8). The observed results demonstrate a pattern of mood and performance decrements in PLA from pre-testing to post-testing without the same decrements observed in the SAL group. One potential explanation for these findings may be related to the study population. The vast majority of the participants in the current study were college students. When scheduling the T1 testing sessions, participants were able to align their starting date with a favorable time in their personal schedule in the immediate future. This allowed for optimal mental and physical preparation for these sessions. Conversely, T2 testing sessions were scheduled to begin 14 days after their T1D1 date resulting in less control of what daily work-, life-, or school-related stressors were occurring during the T2 testing time frame. Prior research involving students at various age levels has demonstrated alterations in physiological functioning, namely cortisol patterns, when faced with high-stakes examinations [53]; however, the studied population involved students aged 6–18 years which may not be applicable to our study population. The findings of the current study introduce an interesting variable to consider when designing studies involving performance testing on college students over time when post-testing session scheduling has no flexibility in comparison with pre-testing scheduling. Interestingly, the ability of salidroside to mitigate mood disturbance and performance deficits in the current study is consistent with its purported role as an adaptogen and protective agent against stress [3,54,55]. In agreement with the current study, other researchers reported a similar phenomenon [26]. In a study involving physical education college students, no change in peak power output was observed between pre and post testing for a group supplementing with *R. rosea*; however, the placebo group did experience a significant decrease in peak power output. Future investigations of adaptogens in college-aged students should directly test this hypothesis. Another potential explanation for the reduction in TTE performance may be a result of the muscle damage protocol performed at T2D1. Even though the muscle damage protocol utilized a different muscle group, it is possible it resulted in residual fatigue leading to decreased TTE performance at T2D3 for the PLA group, but not the SAL group as a result of adaptogenic effects.

### 4.4. Salidroside appears to be well-tolerated

No adverse events were reported as a result of supplementation with SAL. Neither group experienced meaningful changes in comprehensive metabolic panel or complete blood count results. Despite a statistical decrease in serum protein in the SAL group (7.2 g/dL to 6.9 g/dL), values were still within reference laboratory ranges [56]. Further, the SAL group did not demonstrate alterations in resting systolic or diastolic blood pressure. Thus, the evidence from the current study suggests short-term SAL supplementation in healthy young adults at 60 mg (30 mg twice per day) is well tolerated.

### 4.5. Study limitations

A major limitation of the current study was the short duration of supplementation. Approximately two weeks of supplementation is unlikely to be long enough to observe full adaptive responses to SAL supplementation. For example, as an AMPK activator [7,8], SAL may be able to induce mitochondrial biogenesis [57], but any physiological significance of this possibility likely needs more than two weeks of supplementation to observe. Likewise, SAL has been demonstrated to upregulate HIF-1α expression and, subsequently, VEGF leading to enhanced angiogenesis [58]. As with the potential for enhanced mitochondrial biogenesis, the supplementation duration was likely too short to observe any meaningful augmentation of capillary density in skeletal muscle. Lastly, the current study did not control for the menstrual cycle of female participants. Although randomization of female participants should have mitigated the influence of this variable, it is worth mentioning that the current study did not control for its potential influence.

### 4.6. Conclusion and future considerations

In conclusion, short-term dietary supplementation with salidroside at a dose of 30 mg twice daily appears to enhance oxygen uptake during high-intensity aerobic exercise and attenuate muscle damage following resistance exercise in healthy young active adults. Salidroside supplementation may also prevent decreases in exercise performance as well as mitigate development of fatigue-inertia. Future studies should investigate the effects of longer-term supplementation of salidroside at 60 mg per day.

## Supplementary Material

Supplemental Material

## Data Availability

The raw data supporting the conclusions of this article will be made available by the authors upon reasonable request.
