# Supplementary material for: Salidroside and exercise performance in healthy active young adults – an exploratory, randomized, double-blind, placebo-controlled study
Source: J Int Soc Sports Nutr. 2024 Nov 27;21(1):2433744. doi: 10.1080/15502783.2024.2433744 (PMC11610317; doi:10.1080/15502783.2024.2433744)
Supplement: Supplemental Material [file RSSN_A_2433744_SM1150.docx]

# Supplementary Tables

| **Supplementary Table 1.** Comprehensive Metabolic Panel and Complete Blood Count Results | | | | | | | | | | | | | | | |
| --- | --- | --- | --- | --- | --- | --- | --- | --- | --- | --- | --- | --- | --- | --- | --- |
|  |  | **Placebo** | | | | | | | **Salidroside** | | | | | | |
| **Variable** | **Time** | **Mean** | **±** | **SD** | **Median** | **IQR** | | | **Mean** | **±** | **SD** | **Median** | **IQR** | | |
| **Glucose (mg/dL)** | Pre | 92.1 | ± | 5.2 | 92 | 88 | - | 96 | 93.3 | ± | 8.1 | 93 | 89 | - | 98 |
|  | Post | 92.4 | ± | 7.3 | 92 | 88 | - | 98 | 92.2 | ± | 6.7 | 93 | 86 | - | 96 |
| **BUN (mg/dL)** | Pre | 13.6 | ± | 3.4 | 14 | 11 | - | 15 | 13.4 | ± | 3.2 | 13 | 11 | - | 16 |
|  | Post | 15.4 | ± | 3.3 | 15 | 13 | - | 17 | 13.3 | ± | 3.4 | 13 | 11 | - | 15 |
| **Creatinine (mg/dL)** | Pre | 1.0 | ± | 0.2 | 0.99 | 0.86 | - | 1.06 | 1.0 | ± | 0.1 | 1.02 | 0.94 | - | 1.11 |
|  | Post | 1.0 | ± | 0.1 | 0.99 | 0.88 | - | 1.07 | 1.0 | ± | 0.2 | 0.98 | 0.87 | - | 1.07 |
| **eGFR (mL/min)** | Pre | 101 | ± | 14.4 | 104 | 91 | - | 110 | 97.7 | ± | 10.9 | 98 | 88 | - | 107 |
|  | Post | 100.2 | ± | 14 | 98 | 86 | - | 111 | 102 | ± | 13.7 | 101 | 90 | - | 113 |
| **BUN/Creatinine Ratio** | Pre | 14 | ± | 3 | 13 | 12 | - | 16 | 13.2 | ± | 2.8 | 13 | 11 | - | 15 |
|  | Post | 15.8 | ± | 2.9 | 16 | 14 | - | 18 | 13.6 | ± | 3.8 | 14 | 11 | - | 15 |
| **Sodium (mEq/L)** | Pre | 141.5 | ± | 2.4 | 141 | 139 | - | 144 | 141.6 | ± | 2 | 142 | 140 | - | 143 |
|  | Post | 141.1 | ± | 2 | 141 | 140 | - | 142 | 141.3 | ± | 1.9 | 141 | 140 | - | 143 |
| **Potassium (mEg/L)** | Pre | 4.6 | ± | 0.4 | 4.5 | 4.4 | - | 4.8 | 4.4 | ± | 0.4 | 4.4 | 4.2 | - | 4.7 |
|  | Post | 4.5 | ± | 0.3 | 4.5 | 4.3 | - | 4.7 | 4.5 | ± | 0.4 | 4.4 | 4.2 | - | 4.7 |
| **Chloride (mmol/L)** | Pre | 102.4 | ± | 2.1 | 102 | 101 | - | 104 | 102 | ± | 2.2 | 102 | 101 | - | 103 |
|  | Post | 102.8 | ± | 1.5 | 103 | 102 | - | 104 | 102.6 | ± | 2.2 | 102 | 101 | - | 105 |
| **Carbon Dioxide (mmol/L)** | Pre | 22.8 | ± | 1.9 | 23 | 22 | - | 24 | 22.8 | ± | 1.5 | 22 | 22 | - | 24 |
|  | Post | 23.2 | ± | 1.5 | 23 | 22 | - | 24 | 23.1 | ± | 1.5 | 23 | 22 | - | 24 |
| **Calcium (mg/dL)** | Pre | 9.6 | ± | 0.3 | 9.7 | 9.4 | - | 9.8 | 9.7 | ± | 0.4 | 9.7 | 9.3 | - | 10 |
|  | Post | 9.5 | ± | 0.3 | 9.5 | 9.3 | - | 9.6 | 9.6 | ± | 0.4 | 9.6 | 9.4 | - | 9.8 |
| **Protein (g/dL)** | Pre | 7.0 | ± | 0.3 | 7.0 | 6.8 | - | 7.2 | 7.2 | ± | 0.4 | 7.2 | 6.9 | - | 7.4 |
|  | Post | 7.0 | ± | 0.5 | 7.0 | 6.6 | - | 7.2 | 6.9 | ± | 0.4 | 7.0 | 6.7 | - | 7.1 |
| **Albumin (g/dL)** | Pre | 4.8 | ± | 0.3 | 4.8 | 4.6 | - | 5 | 4.8 | ± | 0.3 | 4.9 | 4.6 | - | 5.1 |
|  | Post | 4.7 | ± | 0.3 | 4.7 | 4.6 | - | 4.9 | 4.7 | ± | 0.3 | 4.7 | 4.5 | - | 4.9 |
| **Globulin (g/dL)** | Pre | 2.3 | ± | 0.4 | 2.2 | 2.1 | - | 2.4 | 2.4 | ± | 0.3 | 2.4 | 2.1 | - | 2.5 |
|  | Post | 2.2 | ± | 0.4 | 2.1 | 1.9 | - | 2.5 | 2.3 | ± | 0.2 | 2.3 | 2.2 | - | 2.4 |
| **A/G Ratio** | Pre | 2.2 | ± | 0.4 | 2.1 | 1.9 | - | 2.4 | 2.1 | ± | 0.3 | 2 | 1.9 | - | 2.3 |
|  | Post | 2.2 | ± | 0.4 | 2.2 | 1.8 | - | 2.5 | 2.1 | ± | 0.3 | 2.1 | 1.9 | - | 2.3 |
| **Bilirubin (mg/dL)** | Pre | 0.9 | ± | 1.1 | 0.5 | 0.3 | - | 1.0 | 0.7 | ± | 0.5 | 0.6 | 0.4 | - | 0.8 |
|  | Post | 0.9 | ± | 1.1 | 0.5 | 0.4 | - | 1.0 | 0.6 | ± | 0.4 | 0.5 | 0.3 | - | 0.9 |
| **ALP (IU/L)** | Pre | 72.7 | ± | 13.6 | 70 | 65 | - | 80 | 74.7 | ± | 19.7 | 71 | 66 | - | 83 |
|  | Post | 72.2 | ± | 14.5 | 73 | 60 | - | 83 | 71.4 | ± | 17.7 | 69 | 62 | - | 85 |
| **AST (IU/L)** | Pre | 23.3 | ± | 7.3 | 21 | 18 | - | 26 | 20.9 | ± | 6.1 | 20 | 17 | - | 25 |
|  | Post | 21.5 | ± | 7.5 | 20 | 17 | - | 24 | 20.2 | ± | 6.1 | 19 | 16 | - | 23 |
| **ALT (IU/L)** | Pre | 20.7 | ± | 10.7 | 17 | 14 | - | 22 | 16.7 | ± | 7.1 | 14 | 11 | - | 22 |
|  | Post | 18.7 | ± | 9.6 | 15 | 12 | - | 23 | 17.1 | ± | 7.6 | 14 | 11 | - | 23 |
| **WBC (10^9^/L)** | Pre | 5.5 | ± | 1.3 | 5.4 | 4.6 | - | 5.9 | 5.5 | ± | 1.2 | 5.3 | 4.7 | - | 6.4 |
|  | Post | 5.2 | ± | 1.2 | 5.2 | 4.4 | - | 5.7 | 5.8 | ± | 1.4 | 5.6 | 5.3 | - | 6.2 |
| **RBC (10^12^/L)** | Pre | 4.9 | ± | 0.5 | 4.99 | 4.51 | - | 5.32 | 5 | ± | 0.4 | 5.04 | 4.6 | - | 5.36 |
|  | Post | 4.9 | ± | 0.5 | 4.96 | 4.57 | - | 5.29 | 4.8 | ± | 0.5 | 4.9 | 4.48 | - | 5.12 |
| **Hemoglobin (g/L)** | Pre | 14.5 | ± | 1.5 | 14.7 | 13.5 | - | 15.6 | 14.6 | ± | 1.2 | 14.4 | 13.5 | - | 15.5 |
|  | Post | 14.5 | ± | 1.4 | 14.5 | 13.4 | - | 15.6 | 14.2 | ± | 1.5 | 14.5 | 12.8 | - | 15.3 |
| **Hematocrit (%)** | Pre | 44.8 | ± | 4.1 | 45.5 | 41.9 | - | 48.4 | 44.9 | ± | 3.4 | 44.8 | 42.1 | - | 47.4 |
|  | Post | 44.4 | ± | 4.2 | 44.4 | 41.2 | - | 48.1 | 43.7 | ± | 4.3 | 43.7 | 40.8 | - | 46.3 |
| **MCV (fL)** | Pre | 91.4 | ± | 3.8 | 91 | 89 | - | 94 | 90.4 | ± | 3.6 | 91 | 88 | - | 93 |
|  | Post | 90.8 | ± | 4.0 | 91 | 88 | - | 94 | 90.5 | ± | 3.9 | 91 | 89 | - | 93 |
| **MCH (pg)** | Pre | 29.6 | ± | 1.6 | 29.7 | 28.4 | - | 31 | 29.4 | ± | 1.6 | 29.6 | 28.7 | - | 30 |
|  | Post | 29.5 | ± | 1.6 | 29.5 | 28.5 | - | 30.9 | 29.3 | ± | 1.8 | 29.6 | 28.7 | - | 30.2 |
| **MCHC (g/L)** | Pre | 32.4 | ± | 0.9 | 32.3 | 31.7 | - | 33.2 | 32.5 | ± | 1.1 | 32.7 | 32 | - | 33.3 |
|  | Post | 32.6 | ± | 1 | 32.3 | 32 | - | 33.4 | 32.4 | ± | 1.2 | 32.5 | 31.8 | - | 33.3 |
| **RDW (fL)** | Pre | 12.8 | ± | 1.2 | 12.6 | 11.9 | - | 12.9 | 12.4 | ± | 0.7 | 12.3 | 12 | - | 12.8 |
|  | Post | 12.7 | ± | 0.9 | 12.5 | 12.1 | - | 13 | 12.3 | ± | 0.7 | 12.1 | 11.9 | - | 12.8 |
| **Platelets (10^9^/L)** | Pre | 244.5 | ± | 61.4 | 238 | 206 | - | 274 | 260.1 | ± | 53.5 | 252 | 218 | - | 308 |
|  | Post | 248.4 | ± | 54.3 | 254 | 205 | - | 284 | 266.4 | ± | 65.8 | 261 | 232 | - | 292 |

| **Supplementary Table 2.** Visual Analogue Responses on a Scale from 1 to 10 for Adverse Events | | | | | | | | | | | | | | |
| --- | --- | --- | --- | --- | --- | --- | --- | --- | --- | --- | --- | --- | --- | --- |
|  | **Placebo** | | | | | | | **Salidroside** | | | | | | |
| **Variable** | **Mean** | **±** | **SD** | **Median** | **IQR** | | | **Mean** | **±** | **SD** | **Median** | **IQR** | | |
| **Headache** | 0.58 | ± | 1.08 | 0 | 0 | - | 1.0 | 0.91 | ± | 1.83 | 0 | 0 | - | 1.3 |
| **Fever** | 0.01 | ± | 0.04 | 0 | 0 | - | 0.0 | 0.08 | ± | 0.41 | 0 | 0 | - | 0.0 |
| **Vomiting** | 0.00 | ± | 0.00 | 0 | 0 | - | 0.0 | 0.09 | ± | 0.43 | 0 | 0 | - | 0.0 |
| **Lethargy/Fatigue** | 1.72 | ± | 2.14 | 1 | 0 | - | 2.5 | 1.00 | ± | 1.54 | 0 | 0 | - | 2.5 |
| **Loss of appetite** | 0.37 | ± | 1.00 | 0 | 0 | - | 0.0 | 0.64 | ± | 1.60 | 0 | 0 | - | 0.0 |
| **Insomnia** | 0.57 | ± | 1.61 | 0 | 0 | - | 0.0 | 0.21 | ± | 0.76 | 0 | 0 | - | 0.0 |
| **Depression** | 0.30 | ± | 1.21 | 0 | 0 | - | 0.0 | 0.76 | ± | 1.92 | 0 | 0 | - | 0.0 |
| **Anxiety** | 0.50 | ± | 1.26 | 0 | 0 | - | 0.0 | 0.76 | ± | 1.51 | 0 | 0 | - | 1.0 |
| **Skin Rash** | 0.00 | ± | 0.00 | 0 | 0 | - | 0.0 | 0.00 | ± | 0.00 | 0 | 0 | - | 0.0 |
| **Diarrhea** | 0.22 | ± | 1.04 | 0 | 0 | - | 0.0 | 0.21 | ± | 1.02 | 0 | 0 | - | 0.0 |
| **Dry Mouth*** | 1.46 | ± | 2.25 | 0 | 0 | - | 2.8 | 0.43 | ± | 1.21 | 0 | 0 | - | 0.0 |
| **Dizziness** | 0.50 | ± | 1.24 | 0 | 0 | - | 0.0 | 0.30 | ± | 0.63 | 0 | 0 | - | 0.0 |
| **Shortness of breath** | 0.20 | ± | 0.94 | 0 | 0 | - | 0.0 | 0.17 | ± | 0.84 | 0 | 0 | - | 0.0 |
| **Blurred vision** | 0.00 | ± | 0.00 | 0 | 0 | - | 0.0 | 0.00 | ± | 0.00 | 0 | 0 | - | 0.0 |
| **Fast heart rate/palpitations** | 0.00 | ± | 0.00 | 0 | 0 | - | 0.0 | 0.40 | ± | 1.34 | 0 | 0 | - | 0.0 |
| **Nervousness** | 0.22 | ± | 0.74 | 0 | 0 | - | 0.0 | 0.51 | ± | 1.31 | 0 | 0 | - | 0.0 |
| **Upset stomach/Nausea** | 0.14 | ± | 0.47 | 0 | 0 | - | 0.0 | 0.60 | ± | 1.89 | 0 | 0 | - | 0.0 |
| **Drowsiness** | 0.84 | ± | 1.93 | 0 | 0 | - | 0.4 | 0.22 | ± | 0.63 | 0 | 0 | - | 0.0 |
